# Cardiovascular Risk Assessment in Childhood: Focus on Family History and Lifestyle Habits in the Real-World, Cross-Sectional, Community-Based RISCAPED Study

**DOI:** 10.3390/medicina62101947

**Published:** 2026-10-09

**Authors:** Silvia Alessandra Magrassi, Enrica Bertelli, Costanza Massarino, Enrico Felici, Andrea Secco, Barbara Roviglione, Riccardo Mazzucco, Marinella Bertolotti, Maria Carla Giaccari, Annalisa Roveta, Francesca Cairello, Antonio Maconi

**Affiliations:** 1 Pediatric and Pediatric Emergency Unit, Children Hospital, IRCCS AOU SS Antonio e Biagio e Cesare Arrigo, 15121 Alessandria, Italy; 2 Department of Research and Innovation (DAIRI), IRCCS AOU SS Antonio e Biagio e Cesare Arrigo, 15121 Alessandria, Italy; 3 Department of Research and Innovation (DAIRI), Azienda Sanitaria Locale AL, 15121 Alessandria, Italy; 4 Primary Care Pediatrician, Azienda Sanitaria Locale AL, 15121 Alessandria, Italy

**Keywords:** pediatric cardiovascular risk, pediatric obesity, arterial hypertension, primary health care, family behavior, family medical history

## Abstract

*Background and Objectives:* Pediatric cardiovascular risk factors tend to track into adulthood, accelerating the atherosclerotic process. This study aimed to assess the prevalence of cardiometabolic risk factors, integrating anthropometric parameters, central adiposity indices, early growth trajectories, family history, and household lifestyle, in a primary-care pediatric cohort (the RISCAPED study). *Materials and Methods:* A cross-sectional multicenter study was conducted across primary care pediatric practices in the province of Alessandria, Northwest Italy, enrolling children aged 6 to 12 years. Weight status was classified according to World Health Organization 2007 criteria, abdominal adiposity by Waist-to-Height Ratio (WHtR > 0.5), and high blood pressure according to American Academy of Pediatrics percentiles (≥95th percentile). Family lifestyle habits were evaluated via the Family Nutrition and Physical Activity (FNPA) screening tool. Factors independently associated with high blood pressure and excess weight were identified using multivariable logistic regression models. *Results:* Among 479 children with complete anthropometric data (median age: 9.0 years), 42.3% presented with excess weight (21.9% overweight, 20.4% obesity). WHtR increased significantly across weight groups (median 0.44, 0.46 and 0.50, respectively; *p* < 0.001). Previously undiagnosed high blood pressurewas identified in 7.1% of the cohort, with a significant systolic blood pressure gradient (*p* < 0.001). Rapid weight gain during early infancy was significantly more frequent in children with obesity (10.6% vs. <5.3%, *p* = 0.025). In multivariable models, a family history of thrombophilic disorders (OR 3.16, 95% CI 1.27–7.41) and a less favourable FNPA score (OR 0.94 per point, 0.89–0.99) were independently associated with high blood pressure; a family history of hyperglycemia was associated overweight/obesity (OR 1.71, 1.06–2.78), and a family history of obesity wa associated with childhood obesity (OR 1.71, 1.02–2.88). *Conclusions:* Cardiovascular risk factors emerge early during primary school age, with central adiposity and silent high blood pressure manifesting prior to clinical disease. Multi-generational family history emerged as a consistent marker of risk, supporting routine multi-domain cardiovascular screening in primary pediatric healthcare.

## 1. Introduction

Cardiovascular diseases (CVD) represent a major public health problem worldwide, ranking as the leading cause of death both globally and in Italy. CVD are also the leading cause of premature mortality (<70 years) from non-communicable diseases (NCDs) worldwide, with an increasing burden of cases among young individuals [1].

Over recent decades, cardiovascular risk in the pediatric age group has become a critical public health issue owing to the rising prevalence of obesity, arterial hypertension, and dyslipidemia already during childhood and adolescence. Scientific evidence demonstrates that cardiovascular risk factors present in childhood tend to persist into adulthood, contributing significantly to the early development of atherosclerotic cardiovascular disease (CVD) [2,3]. Indeed, CVD risk originates from the combination of prenatal, childhood, and adult risk factors: maternal health, genetic predisposition, inadequate fetal nutrition, rapid postnatal growth, and the first 1000 days of life are key determinants. Cardiovascular risk is in fact a dynamic process with a cumulative effect over the life course, a continuum that gradually leads to endothelial damage, vascular and myocardial remodeling, and atherosclerotic processes [4].

Excess weight is reaching alarming levels among younger generations. In Italy, 9.8% of primary-school children (aged 8–9 years) have obesity and 19% are overweight [5]; in Europe, 11% of primary-school children (aged 6–9 years) have obesity and 25% are overweight [6], in the United States, 19.7% of children and adolescents aged 2–19 years present with obesity [7]; and the prevalence of overweight and obesity in childhood remains elevated even in lower-income countries [8]. Unless targeted interventions are implemented at an early age, excess weight tends to persist from childhood into adulthood [2], facilitating the onset of multiple cardiometabolic risk factors.

Concurrently, the incidence of ischemic cardiovascular events in individuals under 50 years of age is rising, in close association with the early spread of cardiometabolic risk factors [9]. To mitigate these trajectories, early identification, personalized interventions, and vigilant growth monitoring are essential [4]. The Italian Society of Pediatrics (SIP) emphasizes that prevention should aim to identify the main cardiovascular risk factors early in individual children [10]. Although metabolic Syndrome (MetS) comprises a cluster of interconnected conditions, including obesity, hypertension, dyslipidemia, and hyperglycemia, the American Academy of Pediatrics (AAP) recommends shifting the clinical focus from the formal diagnosis of MetS toward the systematic screening of individual cardiovascular risk factors [11]. In particular, pediatric arterial hypertension is a frequently underdiagnosed condition [3,12] and represents the modifiable risk factor most strongly associated with premature mortality [13]. Alongside traditional clinical parameters, a central role is played by a positive family history of premature ischemic heart disease [14]. In recent years, the literature has broadened the concept of pediatric cardiovascular risk to include lifestyle- and household-related factors [2,15]; accordingly, the Italian Society of Preventive and Social Pediatrics (SIPPS) identifies dietary and behavioral intervention as the cornerstone of prevention and therapy [16].

The observational RISCAPED study is set against this background: a cross-sectional study conducted in the province of Alessandria, whose primary objective is to describe the distribution and prevalence of potential cardiovascular risk factors in the resident pediatric population. Secondary objectives were to assess the association between weight status and hemodynamic and anthropometric parameters, and to identify factors associated with high blood pressure, overweight and obesity through multivariable analysis. Its multidimensional approach reflects the recommendations of major scientific societies [12,17] and is aimed at defining more effective, family-centered prevention strategies in the Italian pediatric setting [10,16]. We hypothesized that cardiovascular risk factors would already be present in school-aged children in this primary care population, and that they would co-occur in the same children in association with excess weight and a positive family history.

## 2. Materials and Methods

### 2.1. Study Design and Population

The present study is based on data from the RISCAPED (Cardiovascular Risk Screening in Developmental Age) project, an observational, cross-sectional, multicenter, non-profit study conducted at the University Hospital of Alessandria (“SS. Antonio e Biagio e Cesare Arrigo” Children’s Hospital) and the Local Health Authority (ASL) of Alessandria, Piedmont, Italy.

The study population comprises a cross-sectional cohort of 501 school-aged children aged 6 to 12 years resident in the province of Alessandria. Eligibility criteria were age between 6 and 12 years, residence in the province of Alessandria, and willingness of parents or legal guardians to provide written informed consent. No exclusion criteria were applied, so that the cohort would reflect the unselected population attending routine pediatric visits. Participants were recruited through a network of Primary Care Pediatricians of the Province. Of the 32 pediatricians who agreed to take part, 26 (81.3%) enrolled at least one participant; the number of patients enrolled ranged from 8 to 24 per practice. Patients were enrolled directly from the community setting during routine pediatric care between September 2023 and September 2024. Written informed consent was obtained from parents or legal guardians prior to enrollment. The study protocol was approved by the competent Ethics Committee and conducted in accordance with the Declaration of Helsinki. For 22 subjects, clinical data were incomplete. Consequently, complete-case analyses based on standardized percentiles (including weight classification) refer to 479 subjects.

### 2.2. Data Collection Procedures

Data collection was structured using a multidimensional protocol divided into two sections (medical and parental), administered during routine pediatric visits.

Data were subsequently recorded and managed using the REDCap platform (Research Electronic Data Capture, version 14.0.31; Vanderbilt University, Nashville, TN, USA).

The medical section, completed by primary care pediatricians, recorded anthropometric parameters (body weight, height, waist circumference) and family and personal medical history. Blood pressure was measured during the same visit with a validated oscillometric device and an appropriately sized cuff, with the child seated after a period of rest and the arm supported at heart level. Three consecutive readings were obtained, and the reading judged most technically reliable by the examining pediatrician was retained for analysis. Systolic and diastolic values were subsequently converted into sex-, age- and height-specific percentiles according to the Pediatric Blood Pressure Percentile Tool for Clinical Decision Support devoleped by Martin B. et al., 2022 using the *pedbp* package of R software [18].

Excessive weight gain in the first months of life was recorded by the primary care pediatrician as part of the personal medical history, on the basis of the growth measurements documented in the child’s health records. Pediatricians were instructed during study training to apply the WHO Child Growth Standards for children aged 0–5 years, corresponding to a BMI-for-age z-score between +1 and +2.

The parental section investigated lifestyle and domestic behavioural habits using the Family Nutrition and Physical Activity (FNPA) screening tool, completed by a parent or legal guardian during the same visit [19]. The FNPA was developed to identify family-level behaviours and routines that may predispose children to excess weight, and comprises 20 items grouped into 10 domains: family meals, family eating practices, food choices, beverage choices, restriction and reward, screen time, healthy environment, family activity, child activity, and family schedule and sleep routine. Each item is scored on a four-point scale, with higher values denoting a more favourable practice. The total score ranges from 20 to 80, higher scores indicating a more favourable family nutrition and physical activity environment. No cut-off values distinguishing healthy from unhealthy home environments have been established, and the score was therefore analysed as a continuous variable and, for descriptive purposes, in tertiles. The tool was originally developed and preliminarily validated as an easy-to-use instrument assessing family environmental and behavioural factors predisposing a child to overweight [19], and its predictive validity was subsequently demonstrated in young children [20]. Construct validity has been confirmed in first- and tenth-grade samples, with stronger utility in younger children [21], and FNPA scores have been associated with obesity severity in youth referred to weight management [22].

### 2.3. Clinical Indicators and Reference Standards

Weight status was evaluated using Body Mass Index (BMI: weight/height^2^, kg/m^2^), converted to sex- and age-specific percentiles and z-scores according to the World Health Organization (WHO) 2007 Growth Reference for children aged 5–19 years. Participants were classified into three weight categories: neither overweight/obesity (<85 percentile), overweight (>85 and ≤97 percentile), and obesity (>97 percentile). Central adiposity was assessed using the Waist-to-Height Ratio (waist circumference/heigh), with abdominal obesity defined as a WHtR > 0.5.

Systolic (SBP) and diastolic (DBP) blood pressure values were converted into percentiles according to the American Academy of Pediatrics (AAP) Clinical Practice Guidelines, standardized for sex, age, and height percentiles. High blood pressure was defined as SBP and/or DBP ≥ 95 percentile.

In the absence of birth dates recorded in months, decimal age for percentile calculation was set at the midpoint of the year (e.g., 7.5 years for 7-year-old children). The total FNPA score was calculated as the sum of all 20 individual items (score range: 20–80) and additionally stratified into tertiles to evaluate behavioral risk gradients.

### 2.4. Statistical Analysis

Continuous variables were summarized as median and interquartile range (IQR), whereas categorical variables were summarized as absolute frequencies and percentages. Continuous variables were compared across the three weight-status groups (neither overweight/obesity, overweight, and obesity) using the Kruskal–Wallis test. In case of statistical significance, post-hoc pairwise comparisons were performed using Dunn’s test with Holm correction for multiple comparisons. Categorical variables were compared across the three weight-status groups using the chi-square test or Fisher’s exact test, as appropriate, according to the expected cell frequencies. In case of statistical significance, post-hoc pairwise comparisons were performed using chi-square tests, or Fisher’s exact test, with Holm correction for multiple comparisons.

Overweight was defined as a BMI percentile >85th and ≤97th, whereas obesity was defined as a BMI percentile >97th. High blood pressure was defined as a systolic and/or diastolic blood pressure percentile ≥95th. The Family Nutrition and Physical Activity (FNPA) score was calculated as the sum of the individual items and was additionally categorized into tertiles for descriptive analyses.

Multivariable binary logistic regression models were fitted to identify factors associated with high blood pressure, overweight/obesity, and obesity. Candidate predictors were selected a priori based on their clinical relevance and were considered for inclusion in the respective models. Variables common to all models included age, sex, ethnicity, previous acute myocardial infarction, arrhythmias, congenital heart disease, sudden cardiac death, hypercholesterolemia, dyslipidemia, hyperglycemia, diabetes mellitus, thrombophilic disorders, thyroid disease, prematurity, low or high birth weight, excessive growth, type 2 diabetes, chronic diseases or pharmacological treatments, obstructive sleep apnea, previous neoplastic disease, and the FNPA score.

To avoid incorporating variables that were directly defined or were intrinsically related to the outcome, blood pressure measurements and blood pressure percentiles were not considered candidate predictors in the high blood pressure model. Similarly, BMI, BMI percentile, and BMI z-score were not considered as candidate predictors in the overweight/obesity and obesity models. Other outcome-related variables were excluded from the corresponding models when they represented the same construct as the dependent variable.

For each outcome, forward stepwise variable selection was performed among the prespecified clinically relevant candidate variables using the subset of participants with complete data for all candidate predictors considered in the selection procedurestarting from a null model containing only the intercept. Model selection was based on the Akaike information criterion (AIC), with a penalty parameter of k = 2. Variables retained by the selection procedure were subsequently included in the final multivariable logistic regression model. Associations were reported as odds ratios (ORs) with 95% confidence intervals (CIs), where applicable. As a sensitivity analysis, given the relatively low proportion of participants with high blood pressure, the forward selection procedure for this outcome was repeated using Firth’s penalized logistic regression, with covariates entered based on a penalized likelihood ratio test (*p* < 0.10) rather than AIC, to mitigate concerns regarding overfitting and the instability of AIC-based comparisons under a limited number of events. All tests were two-sided, and a *p*-value <0.05 was considered statistically significant. All statistical analyses were conducted using R software (R Foundation for Statistical Computing, Vienna, Austria, v. 4.4.1).

Generative artificial intelligence (Claude versions Opus 4.5 and Opus 5, Anthropic, San Francisco, CA, USA) was used during the preparation of this manuscript for language editing and for the linguistic reformulation. No artificial intelligence tool was used in the study design, data collection, statistical analysis or interpretation of results, all of which were carried out by the authors.

## 3. Results

### 3.1. Population Characteristics

A total of 501 pediatric subjects were enrolled (479 with complete anthropometric data), with a substantially balanced sex distribution, a median age of 9 years (IQR 8–10), and a predominantly Caucasian composition (91%). Baseline characteristics, overall and by weight category, are reported in Table 1.

The population shows wide anthropometric variability; sex, waist circumference, and waist-to-height ratio across weight categories is detailed in Table 1.

### 3.2. Weight Status and Adiposity

The median BMI percentile in the overall cohort was 76.7 (IQR: 36.3–95.7), indicating a distribution shifted toward higher values compared with the reference population. Applying the WHO thresholds, 276 subjects (57.7%) were classified as neither overweight/obesity, 105 (21.9%) as overweight, and 98 (20.4%) as obese, resulting in an overall prevalence of excess weight of 42.3%. As expected from the classification criteria, median BMI percentiles increased markedly across weight categories, ranging from 41.9 (IQR 20.1–64.6) among subjects without overweight/obesity to 92.7 (IQR 88.6–95.3) among overweight subjects and 99.6 (IQR 98.7–99.9) among those with obesity, highlighting the clear separation between groups.

The waist-to-height ratio shows a progressive increase across weight categories (median 0.44 in neither overweight/obesity, 0.46 in overweight and 0.50 in obese subjects; *p* < 0.001) (Table 1). Post-hoc analyses showed statistically significant differences of waist-to-height ratio between no overweight/obesity and overweight (adjusted *p* = 0.011), no overweight/obesity and obesity (adjusted *p* < 0.001) and between overweight and obesity (adjusted *p* = 0.011).

### 3.3. Blood Pressure and Weight Status

The prevalence of high blood pressure (blood pressure ≥ 95th percentile for sex, age, and height) in the cohort was 7.1%; these subjects were not previously known to be hypertensive. A clear blood pressure gradient was observed across weight categories (Figure 1). Considering systolic and diastolic blood pressure percentiles, a significant increasing trend in the median systolic blood pressure percentile was observed across weight categories, from participants without overweight/obesity to those with overweight and obesity (*p* < 0.001). No significant difference across weight categories was observed for diastolic blood pressure percentiles (*p* = 0.327).

Post-hoc analyses for systolic blood pressure percentiles showed a statistically significant difference only between participants with neither overweight nor obesity and those with obesity (adjusted *p* < 0.001), whereas the comparison between participants with overweight and those with obesity did not reach statistical significance.

### 3.4. Family History and Weight Status

Family history reveals a substantial burden of cardiometabolic risk factors, detailed by weight category in Table 2. Among the items examined, two show a statistically significant association with weight status: family history of overweight/obesity, which rises from 32.8% in neither overweight nor obesity subjects to 51.0% in obese subjects (*p* = 0.006), and family history of sudden death, from 6.9% to 16.5% (*p* = 0.022). Post-hoc pairwise comparisons for family history of overweight/obesity showed a significant difference between the lowest and highest weight categories (no overweight/obesity vs. obesity; adjusted *p* = 0.007), whereas no significant differences were observed between no overweight/obesity and overweight (adjusted *p* = 0.632) or between overweight and obesity (adjusted *p* = 0.512). Similarly, post-hoc analysis for family history of sudden death showed a significant difference only between the two opposite weight categories (adjusted *p* = 0.030). Family history of hypertension (68.0% in obese subjects) and of myocardial infarction (43.3% in obese subjects) shows higher values in the obesity group without reaching statistical significance (*p* = 0.076 and *p* = 0.12, respectively).

Notably, several familial risk factors are present at high rates even among neither overweight nor obesity (family history of myocardial infarction (34.8%), diabetes (36.0%), and arrhythmias (26.2%)), indicating that anamnestic risk is widely distributed regardless of current weight.

### 3.5. Early Growth Factors

Excessive weight gain in the first months of life was significantly more frequent in subjects with obesity (10.6%) than in the other groups (<5.3%; *p* = 0.025) (Table 2). Post hoc analyses revealed that the difference was statistically significant only between the overweight and obesity groups (adjusted *p* = 0.044), whereas all other pairwise comparisons failed to reach statistical significance. Rapid weight gain is therefore confirmed as strongly associated with current weight status; as this is a parent-reported historical variable in a cross-sectional study, the association is consistent with, but does not demonstrate, a causal relationship. Among perinatal factors, no significant association emerged with prematurity, low birth weight (defined as <3rd percentile), or high birth weight. Other clinical comorbidities (type 2 diabetes, dyslipidemia) were very rare in all groups (~1%), consistent with a young cohort in a preclinical phase.

### 3.6. Lifestyle and FNPA Score

Lifestyle was assessed with the FNPA questionnaire in the 443 participants with complete responses. The distribution of the three weight categories differed significantly across FNPA score tertiles (*p* = 0.012): the prevalence of obesity was lowest in the highest tertile (18.2% vs. 22.4% in the lowest), although the pattern was not fully monotonic, as the middle tertile showed the highest prevalence of overweight (31.8%). When considering the score continuous, the median FNPA was similar across weight categories (64, 63, and 62 in the neither overweight nor obesity, overweight, and obesity groups respectively; *p* = 0.12). Item-level responses are reported in Table S1. Among the 20 individual items, only one differed significantly across weight categories: screen use in the child’s bedroom, reported at least occasionally by 53.9% of children with obesity versus 38.6% of those with overweight and 36.4% of those with neither overweight nor obesity (*p* = 0.039); in post-hoc pairwise comparisons this difference was significant only between children with neither overweight nor obesity and those with obesity (adjusted *p* = 0.008), while the comparison between overweight and obesity was borderline (adjusted *p* = 0.067). Family provision of opportunities for physical activity was numerically more frequent among children with neither overweight nor obesity (40.7% answering “very often/always”) than among those with overweight (35.6%) or obesity (34.8%), without reaching statistical significance (*p* = 0.11). Independently of weight status, the questionnaire identified several widespread unfavourable behaviours: only 30.9% of children met the recommendation of less than two hours of daily screen time, 46.7% ate in front of the television often or very often, 26.0% skipped breakfast at least occasionally (17.2% never or almost never), and only 33.4% consumed fruit and vegetables very often or always.

### 3.7. Factors Independently Associated with High Blood Pressure, Overweight/Obesity and Obesity

Multivariable logistic regression models were used to identify factors independently associated with high blood pressure, overweight/obesity, and obesity. Table 3 presents the final models, reporting ORs, 95% CIs and *p*-values for all variables retained in the model.

In the multivariable analyses derived from forward feature selection, different factors were found to be independently associated with high blood pressure, overweight/obesity, and obesity. For high blood pressure, male sex was independently associated with a lower risk (OR 0.36; 95% CI 0.15–0.79; *p* = 0.014), whereas a family history of thrombophilic disorders was associated with a higher risk (OR 3.16; 95% CI 1.27–7.41; *p* = 0.010); a higher FNPA score, indicating a more favourable family nutrition and physical activity environment, was also independently associated with a lower risk of high blood pressure (OR 0.94 per point; 95% CI 0.89–0.99; *p* = 0.027), while a family history of dyslipidemia was retained in the model without reaching statistical significance (OR 0.48; 95% CI 0.15–1.23; *p* = 0.2). For overweight/obesity, family history of impaired glucose tolerance or hyperglycemia was independently associated with the outcome (OR 1.71; 95% CI 1.06–2.78; *p* = 0.028), while family history of sudden death (OR 1.91; 95% CI 0.97–3.85; *p* = 0.063), low birth weight (OR 0.43; 95% CI 0.16–1.03; *p* = 0.070), and the FNPA score (OR 0.97; 95% CI 0.95–1.00; *p* = 0.070) showed borderline associations. For obesity, family history of overweight/obesity was the only factor independently associated with the outcome (OR 1.71; 95% CI 1.02–2.88; *p* = 0.042), whereas family history of sudden death showed a non-significant positive association (OR 1.88; 95% CI 0.88–3.84; *p* = 0.092).

### 3.8. Sensitivity Analysis

Given the low number of high blood pressure events (7.1%), a sensitivity analysis was performed using Firth’s penalized logistic regression to address potential overfitting and instability associated with standard AIC-based selection and logistic regression model (Table S2). Three of the four covariates identified in the primary model (i.e.,: sex, thrombophilic alterations, and FNPA score) were retained also in this model, with comparable direction and magnitude of association (male sex: OR 0.37 95% CI 0.16–0.79; thrombophilic alterations: OR 2.91 95% CI 1.21–6.55; FNPA score: OR 0.94 95% CI 0.90–0.99). Family history of dyslipidemia, additionally retained in the primary model, narrowly failed to reach the pre-specified entry threshold in this sensitivity analysis (*p* = 0.162). Overall, the substantial agreement between the two models supports the robustness of the main findings.

## 4. Discussion

The results of the RISCAPED study indicate that cardiovascular risk is already widely present at school age and manifests as an aggregation of multiple factors, as it does in adults. The pathophysiological model of the cardiovascular continuum suggests that damage begins early in life and progresses across the lifespan, positing a cumulative effect of risk factors over time [2,4]. In line with Calcaterra et al., according to whom the first 1000 days of life represent a critical window for the programming of cardiometabolic risk [4], our data show that a substantial proportion of subjects already carry risk factors at school age. These observations are consistent with the early origin of the atherosclerotic process, although the cross-sectional design does not allow this temporal sequence to be demonstrated.

### 4.1. Weight Status and Central Adiposity

Data show an upward shift of the BMI distribution, with a median at the 76.7th percentile and an overall prevalence of excess weight of 42.3% (overweight 21.9%, obesity 20.4%). This pattern is consistent with what has been described in the literature as a rightward shift in the pediatric populations of industrialized countries [23] and with WHO global epidemiological trends [2,24]; the cross-sectional design, however, does not allow this shift over time to be documented directly. The high concentration above the 97th percentile indicates a non-negligible proportion of severe obesity, which is associated with early metabolic complications (insulin resistance, type 2 diabetes, hepatic steatosis) [25]. Considering the tracking of BMI from childhood into adulthood [26], these data support early, large-scale interventions rather than interventions restricted to high-risk subjects only.

Consistently, the waist-to-height ratio increases progressively across weight categories (median 0.44 in neither overweight nor obesity, 0.46 in overweight, and 0.50 in obese subjects; *p* < 0.001). This indicator, a reliable proxy of central adiposity and complementary to BMI [27], reaches the risk threshold of 0.5 [28] in the obesity group and approaches it already in the overweight group: this finding signals potentially relevant visceral adiposity from the earliest stages of excess weight and supports the integration of additional anthropometric measures into pediatric clinical practice [24].

### 4.2. Blood Pressure

Systolic blood pressure percentiles increased significantly across weight categories (*p* < 0.001), whereas no significant difference was observed for diastolic percentiles (*p* = 0.327). In post-hoc comparisons, systolic percentiles differed only between children with neither overweight nor obesity and those with obesity, while children with overweight did not differ from those without excess weight, suggesting a threshold effect at the level of obesity rather than a graded association. This pattern, together with a 7.1% prevalence of high blood pressure in previously undiagnosed children, outlines a cardiovascular risk profile that is already detectable in childhood. Evidence indicates that an increasing BMI z-score is associated with a higher risk of hypertension, arterial stiffness, and endothelial dysfunction [2,29], confirming the value of blood pressure as an early marker. In line with this evidence, international guidelines recommend routine blood pressure measurement in childhood, with attention to subjects with excess weight, starting from 3 years of age and earlier in at-risk categories [30].

### 4.3. Family History

Family history reveals a substantial burden of cardiometabolic risk factors, which tends to increase as weight status worsens. Among the items examined, two show a statistically significant association with weight status: family history of overweight/obesity (from 32.8% in neither overweight nor obesity to 51.0% in obese subjects; *p* = 0.006) and family history of sudden death (from 6.9% to 16.5%; *p* = 0.022). Family history of hypertension and of myocardial infarction shows higher values in the obesity group without reaching significance. This picture supports the hypothesis of an intergenerational transmission of risk, mediated by genetic predisposition and shared family lifestyles [31,32], and the concept of familial clustering [33]. It is also noteworthy that several familial risk factors are frequent even among neither overweight nor obesity subjects: family history should be systematically included in clinical assessment as a prognostic tool, guiding preventive interventions aimed at the entire family unit. This intergenerational transmission is unlikely to reflect a single mechanism, and more plausibly arises from the interaction between polygenic susceptibility, intrauterine epigenetic programming and a shared domestic environment [34]. The association with a family history of sudden death warrants specific consideration: childhood obesity accelerates subclinical atherosclerosis, left ventricular hypertrophy and early endothelial dysfunction, so that a familial predisposition to acute arrhythmic or vascular events may compound the metabolic and inflammatory burden of obesity into a higher-risk substrate already in youth [35].

### 4.4. Early Growth Factors

Excessive weight gain in the first months of life was significantly more frequent in subjects with obesity (10.6% vs. <5.3%; *p* = 0.025): rapid weight gain is confirmed as strongly associated with current weight status, in line with its recognized role in the literature [4,36]. As this is a parent-reported historical variable in a cross-sectional study, the association is consistent with, but does not demonstrate, a causal relationship. The higher prevalence of elevated BMI (*p* < 0.001) is consistent with tracking, whereby excess weight tends to consolidate along the growth trajectory. Low birth weight, by contrast, was not associated with excess weight and was numerically more frequent in neither overweight nor obesity subjects (8.8% vs. 2.9% in the overweight group; *p* = 0.092). This result should be interpreted with caution: the absolute number of low-birth-weight children was very small (32 in the whole cohort), and low birth weight was defined here as <3rd percentile, a stricter threshold than the <10th percentile commonly used in the literature. This difference in definition and in the resulting prevalence limits any direct comparison with studies reporting an association between low birth weight and cardiometabolic risk.

Some comorbidities (OSAS, chronic diseases, metabolic alterations) appear more frequent in the excess-weight groups, although without statistical significance. These findings identify the first months of life as a critical window for prevention and suggest monitoring of growth curves, attention to rapid weight gain, and early family nutritional education, with follow-up in the first 12–24 months to reduce comorbidities for mitigating future morbidity and mortality [29].

### 4.5. Lifestyle and FNPA Score

The role of non-traditional factors, such as the family environment and behaviors, in pediatric cardiovascular risk is increasingly recognized [37,38]. In our cohort, a more favorable FNPA profile was associated with a lower prevalence of obesity across score tertiles, although the score did not discriminate against current weight status at the individual level. This is plausible for a distal, family-level factor assessed cross-sectionally in a young, largely preclinical cohort: identified by longitudinal studies as one of the main modifiable determinants of childhood obesity [24,39], the FNPA is best read here as a descriptor of the family context and a candidate factor to be confirmed longitudinally, rather than as a marker of present weight status. The cross-sectional design and the self-reported nature of the data reinforce this cautious interpretation. The substantial homogeneity of reported behaviours across weight categories is consistent with the multifactorial aetiology of childhood obesity, in which genetic susceptibility, metabolic factors, socioeconomic determinants and the risk-obesity environment act alongside domestic habits [23,40]; parent-reported measures may additionally be affected by social desirability bias [41], further attenuating the association between declared lifestyle and adiposity. The single item that did differ across weight categories, screen use in the bedroom, is nonetheless a concrete and modifiable target with recognised implications for sedentary behaviour and sleep hygiene. Therefore, strict parental monitoring of screen time (<2 h a day) [42,43,44,45] and snacks (ultra-processed foods, fast food, and sugar-sweetened beverages) [46] is recommended in the literature.

Beyond dietary and screen-related habits, physical fitness and activity levels in childhood carry additional and independent predictive value for future cardiovascular risk, over and above traditional risk factors including BMI [47]. Aerobic exercise, or the combination of aerobic and resistance training, appears to be the most effective modality for reducing body fat and controlling obesity and its cardiometabolic complications [48], and family-based interventions may enhance adherence and sustainability.

Read in this light, the FNPA is best positioned as a primary screening instrument for the domestic environment rather than as a proxy for current weight status, a role that is clinically meaningful given that clusters of childhood risk factors translate into measurable cardiovascular events in adulthood [49], and that current guidance recommends early, systematic and non-stigmatising assessment of the home environment within family-centred intensive health behaviour and lifestyle treatment [50,51,52].

A recent advancement is the approval of glucagon-like peptide-1 receptor agonists (GLP-1RAs) for children and adolescents with severe obesity, liraglutide from 6 years and semaglutide from 12 years of age, when lifestyle and dietary therapy alone prove insufficient [53]. Pharmacological treatment, however, should not be regarded as an alternative to prevention: appropriate lifestyle habits established in early childhood remain the primary strategy, and are intended to prevent the need for pharmacological intervention, which should be reserved for severe or refractory phenotypes and for forms with an underlying genetic basis, within a comprehensive and family-centered plan of care [54].

### 4.6. Factors Independently Associated with High Blood Pressure, Overweight/Obesity and Obesity

Multivariable analysis results provide a better understanding of the factors independently associated with the outcomes. Family history was retained in all three models, with distinct, outcome-specific associations, underscoring the value of systematic family history collection in pediatric care. Similar observations have been reported in pediatric populations, where family history of obesity and cardiometabolic diseases was associated with both the onset and severity of childhood obesity, highlighting the combined contribution of inherited susceptibility and shared family environment to cardiometabolic risk development [55].

The association between high blood pressure and a family history of thrombophilic disorders is more difficult to interpret, as no direct pathophysiological link between inherited thrombophilia and blood pressure regulation in childhood has been established. Given the wide confidence interval and the limited number of hypertensive events, this finding should be regarded as exploratory and requiring confirmation; it may reflect a broader familial cardiovascular predisposition rather than a specific mechanism.

Similarly, the inverse association observed with male sex differs from the pattern generally reported in the literature, in which boys tend to have higher blood pressure levels than girls, particularly with increasing age and during adolescence. However, sex-related differences in blood pressure are strongly influenced by developmental stage. Given the relatively narrow age range of our cohort (6–12 years), our findings may partly reflect differences in the timing of pubertal development, as girls generally enter puberty earlier than boys [52,56].

In contrast, the FNPA score showed a significant inverse association with high blood pressure: each additional point on the score corresponded to a 6% reduction in the odds of high blood pressure (OR 0.94 per point; 95% CI 0.89–0.99), with children from families reporting more favourable nutrition and physical activity habits less likely to be classified as hypertensive.

To our knowledge, this is the first cross-sectional study to report an association between a less favourable FNPA profile and high blood pressure in children. Previous work has examined the FNPA mainly in relation to adiposity and composite cardiovascular risk: Yee et al. found an association with adiposity and overall cardiovascular risk in 10-year-old children, but no significant association with mean arterial pressure considered individually [22]. Our findings provide a rationale for prospective studies investigating whether an unfavourable family environment precedes the development of elevated blood pressure in childhood. The potential clinical relevance of this association is supported by intervention data: a recent systematic review of 27 studies in children and adolescents found that multidimensional programmes combining physical activity, nutrition and family involvement provided the strongest evidence of benefit on blood pressure [57].

Family history of sudden death was retained in both adiposity-related models and showed a consistent positive association, although statistical significance was not reached. It is likely to indicate the presence of broader familial cardiovascular risk which is not completely captured by the variables included in the models. Similarly, low birth weight and the FNPA score were found to be bordering on significant associations with overweight/obesity. Although these findings should be interpreted cautiously, they are consistent with evidence suggesting that pediatric obesity and cardiometabolic risk are shaped by a multifactorial interplay of early-life exposures, lifestyle behaviors, environmental conditions, and social determinants [55].

### 4.7. Strengths and Limitations

This study presents several notable strengths. First, it investigates a large, well-characterized community-based cohort enrolled across primary care pediatric clinics, reflecting real-world pediatric practice rather than a selected hospital outpatient subpopulation. Second, cardiovascular risk was assessed through an integrated, multidimensional protocol combining standardized hemodynamic measures, surrogate markers of central adiposity (WHtR), perinatal and multi-generational family history, and a validated lifestyle assessment tool (FNPA).

Nonetheless, some limitations must be acknowledged. First, the cross-sectional design precludes establishing direct temporal or causal relationships between early factors, behavioral practices, and current cardiometabolic outcomes. Second, BMI and blood pressure percentiles were calculated using the midpoint of the age year rather than each participant’s exact decimal age. Although this approach is unlikely to have substantially affected the overall findings, a small degree of misclassification may have occurred for children whose values were close to percentile-based classification thresholds. Third, personal perinatal history and family medical history were parent-reported, which may introduce recall or reporting bias, although pediatricians assisted in verifying medical records where available. Fourth, no demographic or socioeconomic data beyond age and sex, including indicators of household income, parental education or food insecurity, were collected, so their contribution to the observed associations could not be assessed. Additionally, blood pressure was assessed via standardized triplicated oscillometric measurements during a single office visit. Furthermore, biochemical and metabolic markers (e.g., lipid profile, fasting glucose, insulin) were not systematically collected in this non-invasive screening protocol, as our primary aim was non-invasive, scalable risk stratification in primary care. Finally, the multivariable models were derived using a forward feature selection procedure. Although this approach facilitates the identification of parsimonious models, it is data-driven and may result in unstable variable selection across different samples. Furthermore, the relatively low number of outcome events, particularly for high blood pressure, may have limited statistical power and increased the risk of model overfitting and imprecise effect estimates. Therefore, the identified associations should be considered exploratory, also taking into account the small amount of events, especially for high blood pressure, and require confirmation in independent populations.

## 5. Conclusions

The RISCAPED study confirms that cardiovascular risk originates early and is already widely present at school age, emerging as a multifactorial, cumulative, and dynamic phenomenon. In this cohort, excess weight was highly prevalent (42.3%) and accompanied by an unfavorable cardiometabolic profile: elevated BMI was associated with higher blood pressure and with central adiposity (waist-to-height ratio approaching 0.5 already in overweight children), while family history emerged as the risk factor most consistently associated with high blood pressure, overweight/obesity, and obesity, pointing to a combined role of genetic predisposition and shared family environment. These findings support early intervention on the comorbidities of childhood obesity, although longitudinal studies are necessary to quantify its efficacy on morbidity and mortality and to suggest improvements in the strategy and procedures of the interventions themselves.

Rapid weight gain in the first months of life was confirmed as an early correlate of obesity, reinforcing the concept of early-life programming [4], whereas family lifestyle, captured by the FNPA score, was not associated with current weight status but showed an independent inverse association with high blood pressure, identifying the household environment as a potentially modifiable target already at school age. Notably, a substantial family history of cardiovascular disease was present even among neither overweight nor obesity children, indicating that risk extends well beyond those with obesity. Taken together, these findings delineate a preclinical window of risk in which timely, integrated action can meaningfully alter lifetime disease trajectories [58,59]. Although the cross-sectional design precludes establishing direct causal relationships, these findings lay out the groundwork for identifying priority areas and generating targeted hypotheses for future longitudinal investigations. Beyond the individual family, reducing the burden of childhood obesity also requires society-level action on the structural inequities that shape access to healthy food and to opportunities for physical activity [40].

In the Italian pediatric setting, the FNPA questionnaire may represent a valuable screening tool for the early identification of modifiable lifestyle-related cardiovascular risk factors, helping to detect children at increased risk of developing obesity and high blood pressure. In conclusion, our results support the paradigm shift from the management of established disease toward early, systematic, and family-centered prevention, integrating anthropometric, hemodynamic, and behavioral screening since early childhood to identify modifiable risk factors and mitigate the long-term burden of adult cardiovascular disease.

## Figures and Tables

**Figure 1:**
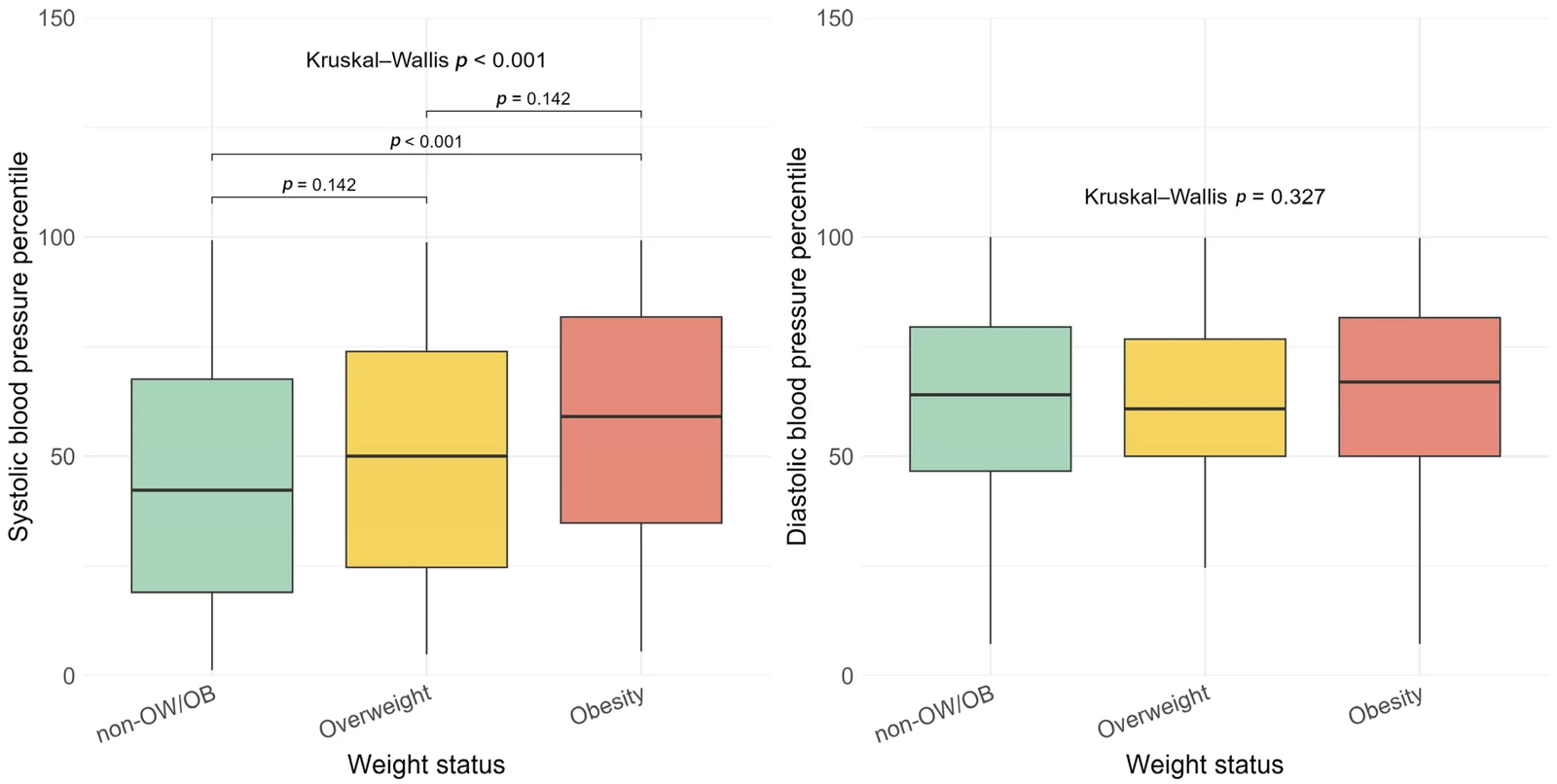
Distribution of systolic and diastolic blood pressure percentiles by weight category. Box plots show the median, interquartile range, and range of systolic (**left**) and diastolic (**right**) blood pressure percentiles in neither overweight nor obesity (non-OW/OB), overweight, and obesity groups. Systolic blood pressure showed a significant gradient across weight categories (*p* < 0.001).

**Table 1:** Demographic, anthropometric, hemodynamic and lifestyle characteristics of the study population, overall and by weight category.

Variable	Total (*n* = 479) ^1^	Neither Overweight/Obesity (*n* = 276) ^1^	Overweight (*n* = 105) ^1^	Obesity (*n* = 98) ^1^	*p* ^2^
Female sex	237 (49.5)	141 (51.1)	53 (50.5)	43 (43.9)	0.5
Age (years)	9.0 (8.0–10.0)	9.0 (8.0–10.0)	9.0 (8.0–10.0)	9.0 (8.0–10.0)	0.8
Waist circumference (cm)	61.0 (56.0–70.0)	59.0 (55.0–66.0)	63.0 (58.0–69.6)	67.5 (58.6–79.0)	<0.001
Missing	4	3	1	0	
Waist-to-height ratio	0.5 (0.4–0.5)	0.4 (0.4–0.5)	0.5 (0.4–0.5)	0.5 (0.5–0.6)	<0.001
Missing	4	3	1	0	
Systolic blood pressure percentile	50.0 (24.2–71.9)	42.3 (19.0–67.6)	50.0 (24.7–73.9)	59.0 (34.8–81.8)	<0.001
Missing	1	1	0	0	
Diastolic blood pressure percentile	63.4 (48.6–79.4)	64.1 (46.7–79.5)	60.8 (50.0–76.8)	67.0 (50.0–81.6)	0.33
Missing	1	1	0	0	
FNPA score	63.0 (59.0–68.0)	64.0 (59.0–69.0)	63.0 (59.0–66.0)	62.0 (57.0–67.0)	0.12
Missing	36	23	4	9	

^1^ *n* (%) for categorical variables; median (interquartile range) for continuous variables. ^2^ Pearson’s Chi-squared test for categorical variables; Kruskal–Wallis rank sum test for continuous variables. FNPA, Family Nutrition and Physical Activity screening tool.

**Table 2:** Family and personal history by weight category.

Characteristic	Overall, *n* = 479 ^1^	Neither Overweight/Obesity, *n* = 276 ^1^	Overweight, *n* = 105 ^1^	Obesity, *n* = 98 ^1^	*p*-Value ^2^
**Family history of…**					
Acute myocardial infarction	169 (35.4)	96 (34.8)	31 (29.5)	42 (43.3)	0.12
Missing	1	0	0	1	
Arrhythmias	129 (27.1)	72 (26.2)	29 (27.6)	28 (29.2)	0.8
Missing	3	1	0	2	
Sudden death	46 (9.6)	19 (6.9)	11 (10.5)	16 (16.5)	0.022
Missing	2	1	0	1	
High blood pressure	288 (60.4)	155 (56.2)	67 (64.4)	66 (68.0)	0.076
Missing	2	0	1	1	
Hypercholesterolemia	245 (51.4)	135 (48.9)	59 (56.7)	51 (52.6)	0.4
Missing	2	0	1	1	
Dyslipidemias	117 (24.8)	65 (24.0)	31 (29.8)	21 (21.6)	0.4
Missing	7	5	1	1	
Overweight/obesity	181 (38.2)	90 (32.8)	42 (40.4)	49 (51.0)	0.006
Missing	5	2	1	2	
Impaired glucose tolerance/hyperglycemia	109 (22.9)	54 (19.7)	33 (31.4)	22 (22.7)	0.052
Missing	3	2	0	1	
Diabetes	168 (35.2)	99 (36.0)	36 (34.3)	33 (34.0)	>0.9
Missing	2	1	0	1	
Thyroid disease	167 (35.2)	89 (32.4)	43 (41.3)	35 (36.5)	0.3
Missing	4	1	1	2	
**Personal history of…**					
Prematurity	41 (8.7)	25 (9.2)	10 (9.6)	6 (6.2)	0.6
Missing	7	5	1	1	
Low birth weight (<3rd percentile)	32 (6.8)	24 (8.8)	3 (2.9)	5 (5.2)	0.092
Missing	5	4	0	1	
High birth weight (>97th percentile)	14 (3.0)	6 (2.2)	4 (3.8)	4 (4.1)	0.5
Missing	5	4	0	1	
Excessive weight gain in the first months of life	25 (5.3)	13 (4.8)	2 (1.9)	10 (10.6)	0.025
Missing	8	3	1	4	

^1^ *n* (%). ^2^ Pearson’s Chi-squared test or Fisher’s exact test, as appropriate.

**Table 3:** Multivariable logistic regression models for high blood pressure, overweight/obesity, and obesity.

Outcome/Variable	OR	95% CI	*p*
High blood pressure ^1^			
Male sex	0.36	0.15–0.79	0.014
Family history of dyslipidemia	0.48	0.15–1.23	0.2
FNPA score	0.94	0.89–0.99	0.027
Family history of trombophilic disorders	3.16	1.27–7.41	0.010
Overweight/obesity ^2^			
Family history of hyperglycemia/IGT	1.71	1.06–2.78	0.028
Low birth weight	0.43	0.16–1.03	0.070
Family history of sudden death	1.91	0.97–3.85	0.063
FNPA score	0.97	0.95–1.00	0.070
Obesity ^3^			
Family history of overweight/obesity	1.71	1.02–2.88	0.042
Family history of sudden death	1.88	0.88–3.84	0.092
Family history of dyslipidemia	0.60	0.32–1.10	0.11

Odds ratios with 95% confidence intervals from three separate multivariable logistic regression models. Only variables retained in each final model are shown. ^1^ Model fit on 410 observations, of which 31 had the event (high blood pressure); ^2^ The model rum on 410 observations, of which 179 had the event (overweight or obesity); ^3^ The model run on 410 observations among which 83 had the event (obesity). CI, confidence interval; FNPA, Family Nutrition and Physical Activity screening tool; IGT, impaired glucose tolerance; OR, odds ratio.

## Data Availability

The data presented in this study are available on request from the corresponding author due to the data are not publicly available because, according to current European and national privacy regulations, the informed consent obtained from parents or legal guardians did not cover public data sharing, and because of the residual risk of re-identification associated with the dataset.

## Supplementary Materials

The following supporting information can be downloaded at: https://www.mdpi.com/article/10.3390/medicina62101947/s1, Table S1: Family Nutrition and Physical Activity (FNPA) screening tool: item-level responses and total score, overall and by weight category; Table S2: Multivariable Firth’s penalized logistic regression model for high blood pressure.

## Abbreviations

The following abbreviations are used in this manuscript:

AAP	American Academy of Pediatrics
AIC	Akaike Information Criterion
BMI	Body mass index
BP	Blood pressure
CI	Confidence interval
CVD	Cardiovascular disease
DBP	Diastolic blood pressure
FNPA	Family Nutrition and Physical Activity
GLP-1RA	Glucagon-like peptide-1 receptor agonist
IGT	Impaired glucose tolerance
IQR	Interquartile range
MetS	Metabolic syndrome
NCD	Non-communicable disease
OR	Odds ratio
OSAS	Obstructive sleep apnea syndrome
SBP	Systolic blood pressure
SIP	Italian Society of Pediatrics (Società Italiana di Pediatria)
SIPPS	Italian Society of Preventive and Social Pediatrics (Società Italiana di Pediatria Preventiva e Sociale)
WHO	World Health Organization
WHtR	Waist-to-height ratio

## References

[1] World Health Organization Cardiovascular Diseases (CVDs). https://www.who.int/news-room/fact-sheets/detail/cardiovascular-diseases-(cvds).

[2] De Blas-Zapata A., Sastre-Albiach J.M., Baixauli-López L., López-Ruiz R., Alvarez-Pitti J. (2025). Emerging Cardiovascular Risk Factors in Childhood and Adolescence: A Narrative Review. Eur. J. Pediatr..

[3] Zhou J., Shan S., Wu J., Song Y., Zhu L., Li Q., Zhang C., Zhu Y., Sheikh A., Rahimi K. (2026). Global Prevalence of Hypertension among Children and Adolescents Aged 19 Years or Younger: An Updated Systematic Review and Meta-Analysis. Lancet Child Adolesc. Health.

[4] Calcaterra V., Mannarino S., Garella V., Rossi V., Biganzoli E.M., Zuccotti G. (2023). Cardiovascular Risk in Pediatrics: A Dynamic Process during the First 1000 Days of Life. Pediatr. Rep..

[5] Indagine Nazionale 2023: Il Convegno Del 10 Maggio 2024. https://www.epicentro.iss.it/okkioallasalute/indagine-2023-convegno-10-maggio-2024.

[6] WHO European Childhood Obesity Surveillance Initiative (COSI): Report on the Sixth Round of Data Collection, 2022–2024. https://www.who.int/europe/publications/i/item/WHO-EURO-2025-11788-51560-78769.

[7] Childhood Obesity Facts|Obesity|CDC. https://www.cdc.gov/obesity/childhood-obesity-facts/childhood-obesity-facts.html.

[8] Bentham J., Di Cesare M., Bilano V., Bixby H., Zhou B., Stevens G.A., Riley L.M., Taddei C., Hajifathalian K., Lu Y. (2017). Worldwide Trends in Body-Mass Index, Underweight, Overweight, and Obesity from 1975 to 2016: A Pooled Analysis of 2416 Population-Based Measurement Studies in 128·9 Million Children, Adolescents, and Adults. Lancet.

[9] Zaheen M., Pender P., Dang Q.M., Sinha E., Chong J.J.H., Chow C.K., Zaman S. (2025). Myocardial Infarction in the Young: Aetiology, Emerging Risk Factors, and the Role of Novel Biomarkers. J. Cardiovasc. Dev. Dis..

[10] Il Cuore Dei Bambini: Le Malattie Cardiovascolari Si Prevengono Nell’infanzia—Società Italiana Di Pediatria. https://sip.it/2024/11/25/il-cuore-dei-bambini-le-malattie-cardiovascolari-si-prevengono-nellinfanzia/.

[11] Sivakoti K. (2024). Adolescent Metabolic Screening. Prim. Care Clin. Off. Pract..

[12] De Ferranti S.D., Steinberger J., Ameduri R., Baker A., Gooding H., Kelly A.S., Mietus-Snyder M., Mitsnefes M.M., Peterson A.L., St-Pierre J. (2019). Cardiovascular Risk Reduction in High-Risk Pediatric Patients: A Scientific Statement From the American Heart Association. Circulation.

[13] Cimmino G., Natale F., Alfieri R., Cante L., Covino S., Franzese R., Limatola M., Marotta L., Molinari R., Mollo N. (2023). Non-Conventional Risk Factors: “Fact” or “Fake” in Cardiovascular Disease Prevention?. Biomedicines.

[14] Taylor C.N., Wang D., Larson M.G., Lau E.S., Benjamin E.J., D’agostino R.B., Vasan R.S., Levy D., Cheng S., Ho J.E. (2023). Family History of Modifiable Risk Factors and Association with Future Cardiovascular Disease. J. Am. Heart Assoc..

[15] Steinberger J., Daniels S.R., Hagberg N., Isasi C.R., Kelly A.S., Lloyd-Jones D., Pate R.R., Pratt C., Shay C.M., Towbin J.A. (2016). Cardiovascular Health Promotion in Children: Challenges and Opportunities for 2020 and Beyond: A Scientific Statement for Healthcare Professionals from the American Heart Association. Circulation.

[16] Società Italiana di Pediatria Preventiva e Sociale (2022). Perché è Importante Rilevare la Pressione nei Bambini. Riv. Ital. Pediatr. Prev. Soc..

[17] Ruan X., Zhu A., Wang T., Sun M., Chen K., Luo M., Li Z., Zou Q., Chen Y., Peng Y. (2025). Global Prevalence of Hypertension in Children and Adolescents Younger Than 19 Years: A Systematic Review and Meta-Analysis. JAMA Pediatr..

[18] Martin B., Dewitt P.E., Albers D., Bennett T.D. (2022). Development of a Pediatric Blood Pressure Percentile Tool for Clinical Decision Support. JAMA Netw. Open.

[19] Ihmels M.A., Welk G.J., Eisenmann J.C., Nusser S.M. (2009). Development and Preliminary Validation of a Family Nutrition and Physical Activity (FNPA) Screening Tool. Int. J. Behav. Nutr. Phys. Act..

[20] Ihmels M.A., Welk G.J., Eisenmann J.C., Nusser S.M., Myers E.F. (2009). Prediction of BMI Change in Young Children with the Family Nutrition and Physical Activity (FNPA) Screening Tool. Ann. Behav. Med..

[21] Peyer K.L., Welk G.J. (2017). Construct Validity of an Obesity Risk Screening Tool in Two Age Groups. Int. J. Environ. Res. Public Health.

[22] Yee K.E., Eisenmann J.C., Carlson J.J., Pfeiffer K.A. (2011). Association between The Family Nutrition and Physical Activity Screening Tool and Cardiovascular Disease Risk Factors in 10-Year Old Children. Int. J. Pediatr. Obes..

[23] Swinburn B.A., Sacks G., Hall K.D., McPherson K., Finegood D.T., Moodie M.L., Gortmaker S.L. (2011). The Global Obesity Pandemic: Shaped by Global Drivers and Local Environments. Lancet.

[24] Phelps N.H., Singleton R.K., Zhou B., Heap R.A., Mishra A., Bennett J.E., Paciorek C.J., Lhoste V.P., Carrillo-Larco R.M., Stevens G.A. (2024). Worldwide Trends in Underweight and Obesity from 1990 to 2022: A Pooled Analysis of 3663 Population-Representative Studies with 222 Million Children, Adolescents, and Adults. Lancet.

[25] Reilly J.J., Kelly J. (2011). Long-Term Impact of Overweight and Obesity in Childhood and Adolescence on Morbidity and Premature Mortality in Adulthood: Systematic Review. Int. J. Obes..

[26] Simmonds M., Llewellyn A., Owen C.G., Woolacott N. (2016). Predicting Adult Obesity from Childhood Obesity: A Systematic Review and Meta-Analysis. Obes. Rev..

[27] Ashwell M., Gibson S. (2016). Waist-to-Height Ratio as an Indicator of ‘Early Health Risk’: Simpler and More Predictive than Using a ‘Matrix’ Based on BMI and Waist Circumference. BMJ Open.

[28] Browning L.M., Hsieh S.D., Ashwell M. (2010). A Systematic Review of Waist-to-Height Ratio as a Screening Tool for the Prediction of Cardiovascular Disease and Diabetes: 0·5 Could Be a Suitable Global Boundary Value. Nutr. Res. Rev..

[29] Valerio G., Di Bonito P., Calcaterra V., Cherubini V., Corica D., De Sanctis L., Di Sessa A., Faienza M.F., Fornari E., Iughetti L. (2024). Cardiometabolic Risk in Children and Adolescents with Obesity: A Position Paper of the Italian Society for Pediatric Endocrinology and Diabetology. Ital. J. Pediatr..

[30] Flynn J.T., Kaelber D.C., Baker-Smith C.M., Blowey D., Carroll A.E., Daniels S.R., De Ferranti S.D., Dionne J.M., Falkner B., Flinn S.K. (2017). Clinical Practice Guideline for Screening and Management of High Blood Pressure in Children and Adolescents. Pediatrics.

[31] Biro F.M., Wien M. (2010). Childhood Obesity and Adult Morbidities. Am. J. Clin. Nutr..

[32] Whitaker R.C., Wright J.A., Pepe M.S., Seidel K.D., Dietz W.H. (1997). Predicting Obesity in Young Adulthood from Childhood and Parental Obesity. N. Engl. J. Med..

[33] Juonala M., Magnussen C.G., Berenson G.S., Venn A., Burns T.L., Sabin M.A., Srinivasan S.R., Daniels S.R., Davis P.H., Chen W. (2011). Childhood Adiposity, Adult Adiposity, and Cardiovascular Risk Factors. N. Engl. J. Med..

[34] Albuquerque D., Nóbrega C., Manco L., Padez C. (2017). The Contribution of Genetics and Environment to Obesity. Br. Med. Bull..

[35] Expert Panel on Integrated Guidelines for Cardiovascular Health and Risk Reduction in Children and Adolescents (2011). Expert Panel on Integrated Guidelines for Cardiovascular Health and Risk Reduction in Children and Adolescents: Summary Report. Pediatrics.

[36] Monteiro P.O.A., Victora C.G. (2005). Rapid Growth in Infancy and Childhood and Obesity in Later Life—A Systematic Review. Obes. Rev..

[37] Tragomalou A., Paltoglou G., Manou M., Kostopoulos I.V., Loukopoulou S., Binou M., Tsitsilonis O.E., Bacopoulou F., Kassari P., Papadopoulou M. (2023). Non-Traditional Cardiovascular Risk Factors in Adolescents with Obesity and Metabolic Syndrome May Predict Future Cardiovascular Disease. Nutrients.

[38] Wang L., Lei J., Wang R., Li K. (2023). Non-Traditional Risk Factors as Contributors to Cardiovascular Disease. Rev. Cardiovasc. Med..

[39] AAP Policy Statements. https://www.aap.org/en/patient-care/institute-for-healthy-childhood-weight/aap-policy-statements-on-obesity/?srsltid=AfmBOoqXOTeGwLqqwh1NxUByuyThB26R6ZjZhcn73X693b2wLMp7FcBe.

[40] Wickramasinghe K., Chatterjee S., Williams J., Weber M.W., Rito A.I., Rippin H., Breda J. (2021). Childhood Overweight and Obesity Abatement Policies in Europe. Obes. Rev..

[41] Birch L.L., Fisher J.O. (1998). Development of Eating Behaviors among Children and Adolescents. Pediatrics.

[42] Fang K., Mu M., Liu K., He Y. (2019). Screen Time and Childhood Overweight/Obesity: A Systematic Review and Meta-Analysis. Child Care Health Dev..

[43] Hill D., Ameenuddin N., Chassiakos Y.R., Cross C., Radesky J., Hutchinson J., Boyd R., Mendelson R., Moreno M.A., Smith J. (2016). Media and Young Minds. Pediatrics.

[44] Horner D., Jahn M., Bønnelykke K., Chawes B., Flensborg-Madsen T., Schoos A.M.M., Stokholm J., Rasmussen M.A. (2025). Screen Time Is Associated with Cardiometabolic and Cardiovascular Disease Risk in Childhood and Adolescence. J. Am. Heart Assoc..

[45] Ghasemirad M., Ketabi L., Fayyazishishavan E., Hojati A., Maleki Z.H., Gerami M.H., Moradzadeh M., Fernandez J.H.O., Akhavan-Sigari R. (2023). The Association between Screen Use and Central Obesity among Children and Adolescents: A Systematic Review and Meta-Analysis. J. Health Popul. Nutr..

[46] Abbasalizad Farhangi M., Mohammadi Tofigh A., Jahangiri L., Nikniaz Z., Nikniaz L. (2022). Sugar-Sweetened Beverages Intake and the Risk of Obesity in Children: An Updated Systematic Review and Dose-Response Meta-Analysis. Pediatr. Obes..

[47] Fraser B.J., Blizzard L., Tomkinson G.R., Dwyer T., Venn A.J., Magnussen C.G. (2026). Added Predictive Value of Childhood Physical Fitness to Traditional Risk Factors for Adult Cardiovascular Disease. Eur. J. Prev. Cardiol..

[48] Headid R.J., Park S.Y. (2020). The Impacts of Exercise on Pediatric Obesity. Clin. Exp. Pediatr..

[49] Corica D., Aversa T., Valenzise M., Messina M.F., Alibrandi A., De Luca F., Wasniewska M. (2018). Does Family History of Obesity, Cardiovascular, and Metabolic Diseases Influence Onset and Severity of Childhood Obesity?. Front. Endocrinol..

[50] Hassapidou M., Duncanson K., Shrewsbury V., Ells L., Mulrooney H., Androutsos O., Vlassopoulos A., Rito A., Farpourt N., Brown T. (2023). EASO and EFAD Position Statement on Medical Nutrition Therapy for the Management of Overweight and Obesity in Children and Adolescents. Obes. Facts.

[51] Genovesi S., Vania A., Caroli M., Orlando A., Lieti G., Parati G., Giussani M. (2024). Non-Pharmacological Treatment for Cardiovascular Risk Prevention in Children and Adolescents with Obesity. Nutrients.

[52] Zhao W., Mo L., Pang Y. (2021). Hypertension in Adolescents: The Role of Obesity and Family History. J. Clin. Hypertens..

[53] Stefater-Richards M.A., Jhe G., Zhang Y.J. (2025). GLP-1 Receptor Agonists in Pediatric and Adolescent Obesity. Pediatrics.

[54] Hampl S.E., Hassink S.G., Skinner A.C., Armstrong S.C., Barlow S.E., Bolling C.F., Edwards K.C.A., Eneli I., Hamre R., Joseph M.M. (2023). Clinical Practice Guideline for the Evaluation and Treatment of Children and Adolescents with Obesity. Pediatrics.

[55] Umano G.R., Bellone S., Buganza R., Calcaterra V., Corica D., De Sanctis L., Di Sessa A., Faienza M.F., Improda N., Licenziati M.R. (2025). Early Roots of Childhood Obesity: Risk Factors, Mechanisms, and Prevention Strategies. Int. J. Mol. Sci..

[56] Li Y., Dong Y., Zou Z., Gao D., Wang X., Yang Z., Dong B., Ma J. (2021). Association between Pubertal Development and Elevated Blood Pressure in Children. J. Clin. Hypertens..

[57] Tiplady C.H., Mynard J.P., Vandeleur M., Ainkaran G., Li S.X., Climie R., Burgner D.P., Dwyer T., Quinlan C., Longmore D.K. (2026). Lifestyle Interventions Addressing Blood Pressure in Children: A Systematic Review. JAMA Pediatr..

[58] Jacobs D.R., Woo J.G., Sinaiko A.R., Daniels S.R., Ikonen J., Juonala M., Kartiosuo N., Lehtimäki T., Magnussen C.G., Viikari J.S.A. (2022). Childhood Cardiovascular Risk Factors and Adult Cardiovascular Events. N. Engl. J. Med..

[59] Candelino M., Tagi V.M., Chiarelli F. (2022). Cardiovascular Risk in Children: A Burden for Future Generations. Ital. J. Pediatr..

