# Supplementary material for: Cardiovascular Risk Assessment in Childhood: Focus on Family History and Lifestyle Habits in the Real-World, Cross-Sectional, Community-Based RISCAPED Study
Source: Medicina (Kaunas). 2026 Oct 9;62(10):1947. doi: 10.3390/medicina62101947 (PMC13706843; doi:10.3390/medicina62101947)
Supplement: Supplementary file 1 [file medicina-62-01947-s001.zip › Table_S1.pdf]

Table S1. Family Nutrition and Physical Activity (FNPA) screening tool: item-level responses and total score, overall and by weight category

Characteristic	Overall, N = 443[1]	Neither overweight nor obesity, N = 253[1]	Overweight, N = 101[1]	Obesity, N = 89[1]	p-value[2]
**1. How often does your child eat breakfast, either at home or at school?**					0.7
1 – Never/almost never	76 (17.2)	42 (16.6)	16 (15.8)	18 (20.2)	
2 – Sometimes	39 (8.8)	24 (9.5)	8 (7.9)	7 (7.9)	
3 – Often	42 (9.5)	22 (8.7)	14 (13.9)	6 (6.7)	
4 – Very often/always	286 (64.6)	165 (65.2)	63 (62.4)	58 (65.2)	
**2. How often does your child eat at least one meal a day with at least one other family member?**					0.072
1 – Never/almost never	0 (0.0)	0 (0.0)	0 (0.0)	0 (0.0)	
2 – Sometimes	5 (1.1)	1 (0.4)	1 (1.0)	3 (3.4)	
3 – Often	36 (8.1)	26 (10.3)	6 (5.9)	4 (4.5)	
4 – Very often/always	402 (90.7)	226 (89.3)	94 (93.1)	82 (92.1)	
**3. How often does your child eat while watching TV (meals and snacks included)? (R)**					0.2
1 – Never/almost never	115 (26.0)	71 (28.1)	25 (24.8)	19 (21.3)	
2 – Sometimes	121 (27.3)	77 (30.4)	25 (24.8)	19 (21.3)	
3 – Often	105 (23.7)	51 (20.2)	26 (25.7)	28 (31.5)	
4 – Very often/always	102 (23.0)	54 (21.3)	25 (24.8)	23 (25.8)	
**4. How often does your family eat fast food? (R)**					0.7
1 – Never/almost never	187 (42.2)	111 (43.9)	39 (38.6)	37 (41.6)	

2 – Sometimes	232 (52.4)	126 (49.8)	59 (58.4)	47 (52.8)	
3 – Often	20 (4.5)	12 (4.7)	3 (3.0)	5 (5.6)	
4 – Very often/always	4 (0.9)	4 (1.6)	0 (0.0)	0 (0.0)	
**5. How often does your family use pre-packaged convenience meals (including frozen ready meals, often designed to be microwaved)? (R)**					0.13
1 – Never/almost never	308 (69.5)	176 (69.6)	64 (63.4)	68 (76.4)	
2 – Sometimes	113 (25.5)	68 (26.9)	29 (28.7)	16 (18.0)	
3 – Often	19 (4.3)	7 (2.8)	8 (7.9)	4 (4.5)	
4 – Very often/always	3 (0.7)	2 (0.8)	0 (0.0)	1 (1.1)	
**6. How often does your child eat fruit and vegetables at meals or as a snack (juices excluded)?**					0.7
1 – Never/almost never	52 (11.7)	29 (11.5)	13 (12.9)	10 (11.2)	
2 – Sometimes	109 (24.6)	60 (23.7)	30 (29.7)	19 (21.3)	
3 – Often	134 (30.2)	74 (29.2)	32 (31.7)	28 (31.5)	
4 – Very often/always	148 (33.4)	90 (35.6)	26 (25.7)	32 (36.0)	
**7. How often does your child drink soda or sugar-sweetened beverages (including regular or diet soda, fruit or vegetable juices, caffeinated energy drinks, sports drinks)? (R)**					0.183
1 – Never/almost never	181 (40.9)	114 (45.1)	36 (35.6)	31 (34.8)	
2 – Sometimes	181 (40.9)	94 (37.2)	45 (44.6)	42 (47.2)	
3 – Often	57 (12.9)	28 (11.1)	15 (14.9)	14 (15.7)	
4 – Very often/always	24 (5.4)	17 (6.7)	5 (5.0)	2 (2.2)	

**8. How often does your child drink low-fat milk at meals or as a snack (including semi-skimmed or skimmed milk, flavoured milk, soy or almond milk)?**					0.4
1 – Never/almost never	258 (58.2)	139 (54.9)	60 (59.4)	59 (66.3)	
2 – Sometimes	93 (21.0)	60 (23.7)	22 (21.8)	11 (12.4)	
3 – Often	56 (12.6)	32 (12.6)	12 (11.9)	12 (13.5)	
4 – Very often/always	36 (8.1)	22 (8.7)	7 (6.9)	7 (7.9)	
**9. How often does your family monitor the amount of candy, chips and cookies your child eats?**					0.7
1 – Never/almost never	21 (4.7)	12 (4.7)	3 (3.0)	6 (6.7)	
2 – Sometimes	46 (10.4)	23 (9.1)	12 (11.9)	11 (12.4)	
3 – Often	133 (30.0)	80 (31.6)	27 (26.7)	26 (29.2)	
4 – Very often/always	243 (54.9)	138 (54.5)	59 (58.4)	46 (51.7)	
**10. How often does your family use candy, ice cream or other sweets as a reward for good behaviour? (R)**					0.6
1 – Never/almost never	330 (74.5)	189 (74.7)	73 (72.3)	68 (76.4)	
2 – Sometimes	90 (20.3)	49 (19.4)	22 (21.8)	19 (21.3)	
3 – Often	16 (3.6)	12 (4.7)	3 (3.0)	1 (1.1)	
4 – Very often/always	7 (1.6)	3 (1.2)	3 (3.0)	1 (1.1)	
**11. How often does your child spend less than 2 hours a day in front of a screen (TV, computer, game consoles or any mobile screen device)?**					>0.9
1 – Never/almost never	76 (17.2)	42 (16.6)	16 (15.8)	18 (20.2)	

2 – Sometimes	116 (26.2)	67 (26.5)	27 (26.7)	22 (24.7)	
3 – Often	114 (25.7)	62 (24.5)	27 (26.7)	25 (28.1)	
4 – Very often/always	137 (30.9)	82 (32.4)	31 (30.7)	24 (27.0)	
**12. How often does your family monitor the amount of time your child spends in front of a screen?**					0.7
1 – Never/almost never	19 (4.3)	10 (4.0)	5 (5.0)	4 (4.5)	
2 – Sometimes	36 (8.1)	24 (9.5)	4 (4.0)	8 (9.0)	
3 – Often	110 (24.8)	61 (24.1)	26 (25.7)	23 (25.8)	
4 – Very often/always	278 (62.8)	158 (62.5)	66 (65.3)	54 (60.7)	
**13. How often does your child spend time in front of a screen in his/her bedroom? (R)**					0.039
1 – Never/almost never	264 (59.6)	161 (63.6)	62 (61.4)	41 (46.1)	
2 – Sometimes	111 (25.1)	60 (23.7)	23 (22.8)	28 (31.5)	
3 – Often	48 (10.8)	24 (9.5)	13 (12.9)	11 (12.4)	
4 – Very often/always	20 (4.5)	8 (3.2)	3 (3.0)	9 (10.1)	
**14. How often does your family provide opportunities for physical activity?**					0.11
1 – Never/almost never	27 (6.1)	8 (3.2)	11 (10.9)	8 (9.0)	
2 – Sometimes	74 (16.7)	43 (17.0)	14 (13.9)	17 (19.1)	
3 – Often	172 (38.8)	99 (39.1)	40 (39.6)	33 (37.1)	
4 – Very often/always	170 (38.4)	103 (40.7)	36 (35.6)	31 (34.8)	
**15. How often does your family encourage your child to be physically active?**					0.11
1 – Never/almost never	18 (4.1)	5 (2.0)	6 (5.9)	7 (7.9)	
2 – Sometimes	41 (9.3)	24 (9.5)	12 (11.9)	5 (5.6)	

3 – Often	153 (34.5)	87 (34.4)	37 (36.6)	29 (32.6)	
4 – Very often/always	231 (52.1)	137 (54.2)	46 (45.5)	48 (53.9)	
**16. How often does your child do physical activity with at least one other family member?**					0.9
1 – Never/almost never	97 (21.9)	55 (21.7)	22 (21.8)	20 (22.5)	
2 – Sometimes	153 (34.5)	82 (32.4)	38 (37.6)	33 (37.1)	
3 – Often	120 (27.1)	73 (28.9)	27 (26.7)	20 (22.5)	
4 – Very often/always	73 (16.5)	43 (17.0)	14 (13.9)	16 (18.0)	
**17. How often does your child do something physically active in his/her free time?**					0.9
1 – Never/almost never	31 (7.0)	14 (5.5)	9 (8.9)	8 (9.0)	
2 – Sometimes	96 (21.7)	55 (21.7)	22 (21.8)	19 (21.3)	
3 – Often	165 (37.2)	94 (37.2)	36 (35.6)	35 (39.3)	
4 – Very often/always	151 (34.1)	90 (35.6)	34 (33.7)	27 (30.3)	
**18. How often does your child take part in organised sports or physical activities with a coach or instructor?**					0.5
1 – Never/almost never	43 (9.7)	27 (10.7)	10 (9.9)	6 (6.7)	
2 – Sometimes	56 (12.6)	26 (10.3)	17 (16.8)	13 (14.6)	
3 – Often	165 (37.2)	92 (36.4)	35 (34.7)	38 (42.7)	
4 – Very often/always	179 (40.4)	108 (42.7)	39 (38.6)	32 (36.0)	
**19. How often does your child follow a regular routine for your child's bedtime?**					0.3
1 – Never/almost never	21 (4.7)	14 (5.5)	3 (3.0)	4 (4.5)	
2 – Sometimes	17 (3.8)	5 (2.0)	7 (6.9)	5 (5.6)	
3 – Often	92 (20.8)	51 (20.2)	24 (23.8)	17 (19.1)	

4 – Very often/always	313 (70.7)	183 (72.3)	67 (66.3)	63 (70.8)	
**20. How often does your child get enough sleep at night?**					0.6
1 – Never/almost never	4 (0.9)	4 (1.6)	0 (0.0)	0 (0.0)	
2 – Sometimes	10 (2.3)	4 (1.6)	2 (2.0)	4 (4.5)	
3 – Often	70 (15.8)	39 (15.4)	18 (17.8)	13 (14.6)	
4 – Very often/always	359 (81.0)	206 (81.4)	81 (80.2)	72 (80.9)	
**Total FNPA score, median (IQR)**	**63.0 (59.0, 68.0)**	**64.0 (59.0, 69.0)**	**63.0 (59.0, 66.0)**	**62.0 (57.0, 67.0)**	**0.12**

[1] Item-level data are presented as n (%).

[2] Pearson's Chi-squared test or Fisher's exact test for categorical items; Kruskal–Wallis rank sum test for the total FNPA score. P-values are unadjusted for multiple comparisons.

(R) Reverse-scored item: the response category indicating the most favourable family behaviour is "Never/almost never". For all other items the most favourable category is "Very often/always".

Each item is scored from 1 to 4; the total FNPA score is the sum of the 20 items (range 20–80), with higher scores indicating a more favourable family nutrition and physical activity environment.

Analyses are restricted to the 443 participants with a complete FNPA questionnaire.
