# Supplementary material for: Cardiovascular Risk Assessment in Childhood: Focus on Family History and Lifestyle Habits in the Real-World, Cross-Sectional, Community-Based RISCAPED Study
Source: Medicina (Kaunas). 2026 Oct 9;62(10):1947. doi: 10.3390/medicina62101947 (PMC13706843; doi:10.3390/medicina62101947)
Supplement: Supplementary file 1 [file medicina-62-01947-s001.zip › Table_S2.pdf]

Table S2. Multivariable Firth's penalized logistic regression model for high blood pressure

Outcome/Variable	OR	95% CI	p
Male sex	0.37	0.16–0.79	0.010
FNPA score	0.94	0.89–0.99	0.025
Family history of trombophilic disorders	2.91	1.21-6.55	0.018

Odds ratios with 95% confidence intervals from three separate multivariable logistic regression models. Only variables retained in each final model are shown.

Model fit on 410 observations, of which 31 had the event (high blood pressure)

CI, confidence interval; FNPA, Family Nutrition and Physical Activity screening tool; IGT, impaired glucose tolerance; OR, odds ratio.
